# Tides of emotion: Hepatologists’ role in navigating depression and anxiety in liver disease

**DOI:** 10.1097/HC9.0000000000000741

**Published:** 2025-07-14

**Authors:** Sarah Khan, Vinay Jahagirdar, Elliot B. Tapper

**Affiliations:** 1 Cleveland Clinic Foundation, Rochester, Minnesota, USA; 2 Department of Medicine, Division of Gastroenterology, Hepatology, & Nutrition, Virginia Commonwealth University, Richmond, Virginia, USA; 3 Michigan Medicine, University of Michigan, Ann Arbor, Michigan, USA

Chronic liver disease (CLD) is strongly associated with mood disorders, especially depression and anxiety. Among people living with CLD, the prevalence of depression is 16% and anxiety is nearly 50%, substantially higher than in the general population.^1^ Biological, environmental, and clinical mechanisms have been proposed to explain this observation. First, CLD can cause depression and anxiety through derangements in the gut-liver access, as it increases dysbiosis, gut permeability, and therefore, the burden of inflammation. This impacts the hypothalamic-pituitary-adrenal axis and metabolism of serotonin and glucocorticoids, hence affecting brain function and behavior.^2^ Second, patients with CLD have a higher prevalence of psychosocial and demographic risk factors for mood disorder, such as lower household income and social isolation.^1^ Third, the severity of CLD itself makes it challenging to distinguish pathologic mood from adjustment disorder. Several articles in *Hepatology Communications* improve our understanding of the complex interaction between CLD and mood disorders, and these are further discussed here.

## IMPACT OF MENTAL HEALTH DISORDERS ON LIVER DISEASE

In their seminal review on depression and anxiety in cirrhosis, Zimbrean and Jakab show that mood disorders have a profound impact on the patient’s global health.^1^ Depression predicts poor adherence to therapy. Depression and anxiety lead to both a higher severity of physical symptoms, including fatigue, and poor response to therapeutic plans.^2^^,^^3^ Mood disorders significantly increase the risk of opioid use, suicide, and all-cause mortality across various etiologies of CLD.^2^^,^^4^ This holds true across the spectrum of disease severity. Even among patients with acute-on-chronic liver failure, Sharapuram et al^5^ showed that poor emotional well-being and energy are associated with increased mortality.

## THE CHALLENGE OF MENTAL HEALTH MANAGEMENT IN THE CONTEXT OF SOMATIC SYMPTOMS

Several physical aspects of the disease experience present challenges to coping with and managing cirrhosis. These include the impact of cramps, pruritus, sleep impairment, sexual dysfunction, and fatigue,^6^ all of which can limit patients’ ability to uphold social roles, causing guilt and anguish.^7^ Recently, Zhang et a^8^ showed that chronic pain is a major inciting factor of distress. Compared to matched controls, patients with CLD have disproportionately higher rates of pain and opioid use. Beyond the functional limitations of pain itself, its management is often ineffective and causes adverse events.

## HEPATOLOGISTS’ ROLE IN SCREENING AND MANAGEMENT

As we guide patients through their illness, hepatologists have the best opportunity to provide early interventions and optimize the management of our patients’ psychological distress. Identification is the first step. Several tools have been validated. The Hospital Anxiety and Depression Scale is a widely used screening tool, consisting of a 14-item questionnaire that has recently been validated for use in patients with decompensated cirrhosis.^9^ It is designed to minimize the influence of somatic symptoms, common in this population, which can confound the assessment of anxiety and depression. The Patient Health Questionnaire-9 for depression, and Generalized Anxiety Disorder-7, State-Trait Anxiety Inventory, are other standard instruments that have been employed for screening in patients with CLD.^10^ Regular screening should be conducted at least semi-annually, or more frequently, particularly for patients with a worsening clinical trajectory.^1^

Next, hepatologists must be comfortable offering first-line therapies for mood disorders. These include referral for cognitive behavioral therapy or mindfulness-based stress reduction to identify and change negative thoughts. Several mobile applications are now available to provide structured mindfulness exercises that can be easily integrated into daily routines.^11^ Physical activity and exercise, tailored to the patient’s physical capabilities, can improve mood. These range from yoga or Tai-Chi to resistance exercise. Importantly, it has been shown that illness apprehension was the sole factor independently linked to physical quality of life, even after considering the severity of liver disease, cognitive status, emotional symptoms, and available support resources.^12^ Above all, education, managing expectations, and reframing can help patients reduce anxiety and understand treatment plans more effectively.^13^

Pharmacological interventions are often helpful. Selective serotonin reuptake inhibitors are generally considered first-line treatments for anxiety and depression in patients with CLD. They are well-tolerated and have a low risk of drug-drug interactions. Benzodiazepines carry the risk of precipitating hepatic encephalopathy and should be avoided. Hydroxyzine and melatonin are safer alternatives for managing insomnia. Even if not personally prescribing, hepatology engagement in medication management ensures that treatments are monitored for potential interactions with their existing medications and adjusted for liver function.

An additional key to addressing the emotional burden of cirrhosis is improving its lived experience. A comprehensive approach that includes symptom management, nutritional support, and psychological care can lead to improvements in mental health and quality of life. Proper control of pruritus has been associated with reductions in sleep disturbances and depressive symptoms. Muscle cramps can be addressed with simple interventions such as sips of pickle brine, meditation, or stretching. Physical therapy, cognitive behavioral therapy, acupuncture, massage, and patient education programs can complement pharmacological options for pain management in addition to limited acetaminophen and expanded use of topical treatments (ie, diclofenac or capsaicin). Ensuring adequate nutrition can help reduce fatigue and sarcopenia. Hepatologists must familiarize themselves with the resources available and partner with other specialties. Thereafter, testing, validating, and treating depression and anxiety are needed. Multidisciplinary approaches ensure that the responsibility for patients’ mental health is shared.^14^ Perhaps the most important partnership is with patients’ caregivers, who are themselves at risk of significant stress and burnout. Collaborative care models not only improve patient outcomes but also enhance caregiver well-being, creating a supportive environment that fosters recovery and long-term management.

## FUTURE DIRECTIONS

Hepatologists orchestrate the collaborative care of patients with CLD. Education and training may enhance hepatologists' confidence in managing the psychosocial burden of liver disease. Enhanced training programs should help foster skills to recognize signs of psychological distress, conduct sensitive prognosis conversations, and apply intervention strategies thoughtfully. Future research should focus on quantitative measures to assess the impact of hepatologist-led interventions on patients’ psychological outcomes. This includes longitudinal studies that track the efficacy of both nonpharmacological and pharmacological interventions. Additionally, exploring the dynamics of patient-hepatologist interactions and their effect on patient trust and treatment adherence could provide deeper insights into optimizing care for patients with CLD.

## CONCLUSIONS

**FIGURE 1:**
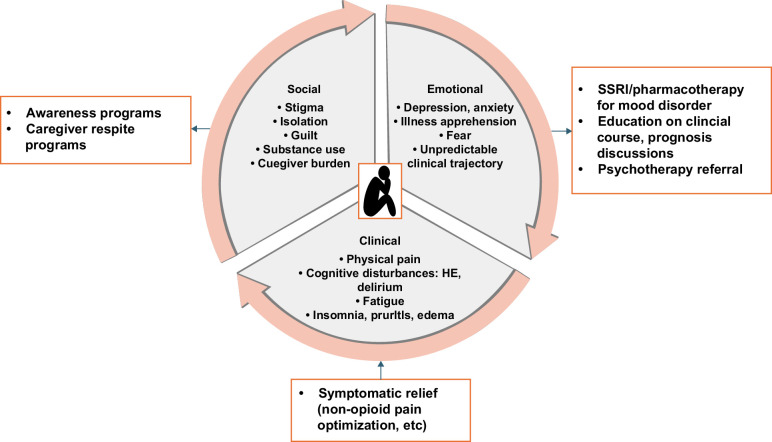
Depression and anxiety in liver disease: symptomatology and mitigating strategies.

The integration of comprehensive psychological care into hepatology practice is crucial for enhancing the quality of life and outcomes for patients with liver disease. Hepatologists must adopt a proactive role in managing not only the physical aspects of CLD but also the psychological complexities that accompany it, both of which profoundly impact emotional well-being. In summary, addressing both the physical and mental health aspects of CLD through a multidisciplinary and proactive approach is essential for improving patient outcomes and quality of life (Figure 1).
